# A progenitor with immature Bergmann glia-like characteristics in the developing cerebellar cortex

**DOI:** 10.3389/fnins.2026.1963374

**Published:** 2026-10-30

**Authors:** Kazuhiko Sawada

**Affiliations:** Department of Nutrition, Faculty of Medical and Health Sciences, Tsukuba International University, Tsuchiura, Japan

**Keywords:** Bergmann glia, cerebellum, ferret, granular neurons, Nestin, Sox2

## Abstract

The cerebellum is an ancient brain component, conserved across mammalian species, that has classically played roles in regulating muscle tone, limb coordination, and equilibrium, as well as in non-motor functions, such as language, cognition, and emotion. Recently, we reported Nestin-expressing cells in the cerebellar cortex of ferret neonates that showed a proliferative potency defined by proliferating cell nuclear antigen (PCNA) immunostaining. We called these cells “internal granular progenitor (IGP).” IGPs were found to be abundant in the internal granular layer adjacent to the Purkinje cell layer and extended GFAP-immunopositive radials progressed through the molecular and external granular layers, forming an immature Bergmann glia-like morphology. The tracking of BrdU-labeling revealed that IGPs differentiated into granular neurons, Bergmann glia, and/or astrocytes. As such, we propose that IGPs may be neural progenitors with an immature Bergmann glia-like morphology that appear in the developing cerebellar cortex, and could serve as another source of granular neurons distinct from external granular progenitors. Further research on IGPs is expected to provide new perspectives for the future investigation of cerebellar corticohistogensis, particularly the difference between motor and non-motor cerebellar regions, and how the abnormal development of IGPs relates to neurodevelopmental disorders associated with non-motor cerebellar function, such as autism spectrum disorder.

## 1 Introduction

The cerebellum is an ancient brain component in mammals that plays roles in the regulation of muscle tone, limb coordination, and equilibrium. In addition to these well-known motor functions, the cerebellum plays a role in non-motor functions, such as language, cognition, and emotion (Leiner et al., 1993; Hu et al., 2008; Rahimi-Balaei et al., 2018; Habas, 2021). Although these non-motor functions are mediated by the cerebellar hemispheres (or the neo-cerebellum), which exhibit gross anatomical and functional asymmetry and have lateralized connections to other brain regions (Hu et al., 2008; Wang et al., 2013; Habas, 2021). The histological and developmental differences between the cerebellar regions responsible for motor and non-motor functions remain unclear. The cerebellar cortex has a simple histoarchitecture organized into three layers: the molecular (ML), Purkinje cell (PCL), and internal granular (IGL) layers (Altman et al., 1997b). Neurons in the cerebellar cortical layers connect to communicate to form intrinsic circuits (Consalez et al., 2021). Granular neurons densely packed in the IGL receive inputs from the spinal cord, brainstem precerebellar nuclei, and vestibular organs via mossy fibers and relay their excitatory signals to Purkinje cells through parallel fibers (Altman et al., 1997b; Consalez et al., 2021). Granular neuron-Purkinje cell excitatory transmission is often amplified by unipolar brush cells and inhibited by GABAergic interneurons, such as molecular layer interneurons (MLIs; basket and stellate neurons) and Golgi neurons (Altman et al., 1997b; Consalez et al., 2021). Purkinje cells inhibit deep cerebellar neurons, which send excitatory outputs to other brain regions, such as the thalamus and red nucleus (Consalez et al., 2021; Rahimi-Balaei et al., 2018). Cerebellar neurons are derived from two distinct germinal epithelial cells in the embryonic cerebellum. One is the ventricular zone in the 4th ventricle roof, identified by *Ptf1a* expression, which generates Purkinje cells and multiple classes of inhibitory interneurons, including MLIs. The other is the upper rhombic lip (uRL), which gives rise to excitatory neurons such as deep cerebellar neurons and unipolar brush cells (Consalez et al., 2021; Rahimi-Balaei et al., 2018). uRL further produces external granular precursors (EGPs) that cover the developing cerebellar surface to form an external granular layer (EGL) (Consalez et al., 2021; Rahimi-Balaei et al., 2018). EGPs proliferate, differentiate into granular neurons through inward migration along Bergmann glial processes, and finally settle in the IGL (Consalez et al., 2021; Rahimi-Balaei et al., 2018). Thus, many studies have documented cerebellar cortical neurogenesis and histogenesis. Herein, this paper highlights a Nestin-expressing neural progenitor appearing in the developing cerebellar cortex in ferrets (Sawada and Kamiya, 2023; Kamiya et al., 2023) and discusses its evolutionary significance and cerebellar function.

## 2 Cerebellar cortical histogenesis in ferrets

Ferrets (*Mustela putorius*), which belong to the order Carnivora, are extensively used as an animal model for studying influenza infection owing to their susceptibility to human influenza viruses (Maher et al., 2004). Recently, ferrets have been increasingly used in neurodevelopmental research because of their gyrencephalic cerebral cortex (Imamura et al., 2024; Kawasaki, 2024; Ross et al., 2024; Sawada, 2026). Additionally, novel insights into cerebellar cortical histogenesis have been obtained in experiments using ferrets. We found cells expressing proliferating cell nuclear antigen (PCNA) with 2 h BrdU-labeling in the IGL adjacent to the PCL in the developing cerebellar cortex of ferret neonates (Kamiya et al., 2024). Notably, PCNA-expressing cells were immunopositive for Nestin (Figure 1A), an intermediate filament selectively expressed in neural stem cells (Lendahl et al., 1990; Omary et al., 2004). These are referred to as internal granular progenitors (IGPs). Cerebellar histogenesis, which forms a three-layered cortex, occurs in ferrets during the early postnatal age and follows a trajectory similar to that of rodents (Christensson et al., 2007; Kamiya and Sawada, 2021). IGPs were temporally found in the ferret cerebellum on postnatal day (PD) 7 (Sawada and Kamiya, 2023), which mirrors the cerebellar corticohistogenesis stage in PD 4 mice (Kamiya and Sawada, 2021). IGPs were also immunopositive for Sox2 (Figures 1A,B) and extended their GFAP-immunopositive processes perpendicularly through the PCL to the ML (Figure 1B). In addition, IGPs were immunopositive for BLBP and S100β (Sawada and Kamiya, 2023), exhibiting immature Bergmann glia-like morphological and immunohistochemical characteristics. The tracking of BrdU labeling revealed that IGPs transitioned to granular neurons, Bergmann glia, and/or astrocytes throughout the differentiative division in normal ferrets (Kamiya et al., 2024), with attenuation of Nestin immunostaining by PD 20 (Sawada and Kamiya, 2023). Therefore, we propose that IGPs function as another source of granular neurons distinct from EGPs.

**Figure 1:**
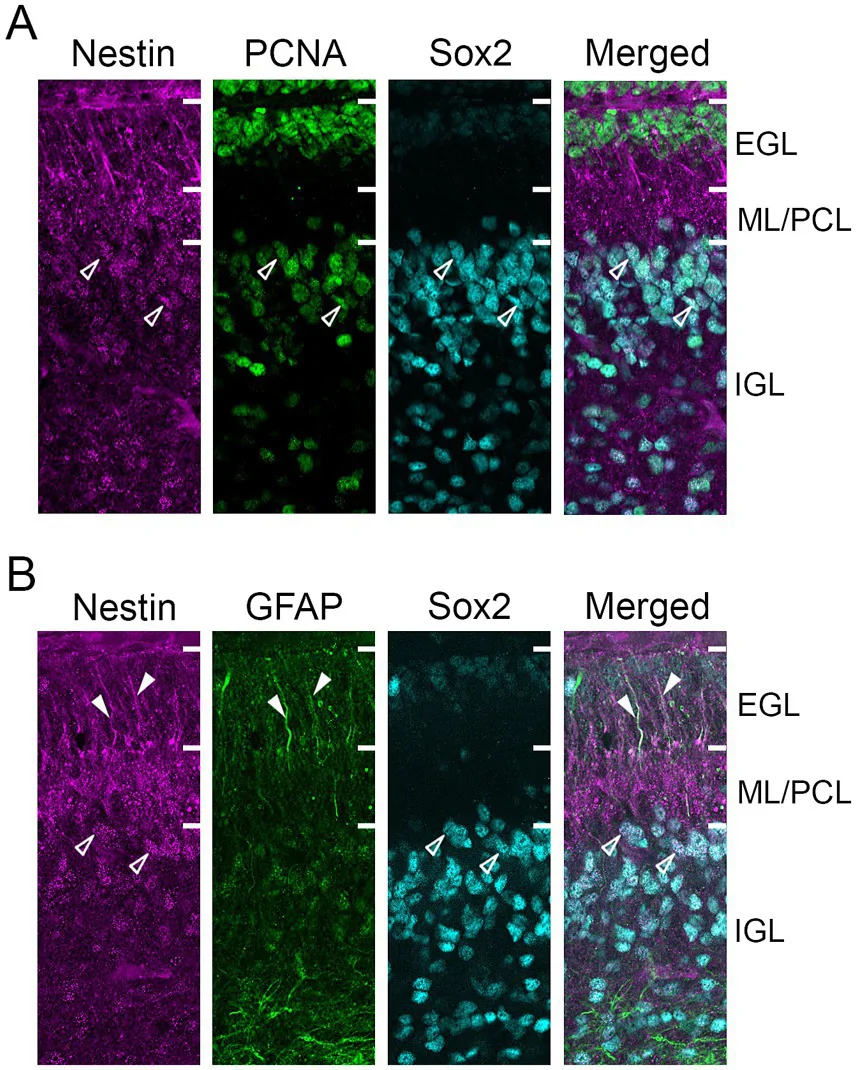
Immunofluorescence for Nestin with neurogenesis and glial markers in developing cerebellar cortex of ferrets on postnatal day 7. **(A)** Triple immunofluorescence staining for Nestin, PCNA and Sox2. Open arrowheads, Nestin-positive internal granular progenitors (IGPs) co-stained with PCNA and Sox2. **(B)** Triple immunofluorescence staining for Nesitin, GFAP and Sox2. Open arrowheads, Nestin-positive IGPs co-stained with GFAP and Sox2; Closed arrowheads, Nestin-positive radial processes co-stained with GFAP. EGL, external granular layer; IGL, internal granular layer; ML, molecular layer; PCL, Purkinje cell layer.

The possibility that IGPs give rise to granular neurons was supported by the expression of myeloid ectopic viral integration site 1 homolog (Meis1) in cells with Bergmann glia-like morphology around the PCL in mouse neonates (Owa et al., 2018). Meis1 is a transcription factor that plays a role in cell cycle exit, granular neuron differentiation, migration towards the IGL, and the subsequent formation of parallel fibers of differentiating granular neurons through the activation of Pax6 (Owa et al., 2018).

Persistent Sox2 expression is a unique characteristic of Bergmann glia. Interestingly, the Bergmann glial lineage expressing Sox2, but not glial marker antigens such as S100β, GFAP, and Olig2, may serve as adult neural progenitors (Ahlfeld et al., 2017). As IGPs also have a lineage to Bergmann glia (Kamiya et al., 2023), the possibility that adult cerebellar neural progenitors originate from an IGP-derived Bergmann glial population should also be addressed.

A recent study by Suyama et al. (2026) reported that Bergmann glia-like progenitors (BGLPs) transiently appear in the cerebellum of mouse neonates. Similar to IGPs, BGLPs have a Bergmann glia-like morphology with the lineage potential to differentiate into MLIs, Bergmann glia and IGL astrocytes; however, their potential to differentiate into granular neurons is unknown (Suyama et al., 2026). Further, Nestin expressing progenitors (NEPs) have been identified in the cerebellum of mouse neonates (Wojcinski et al., 2017); these cells primarily produce astrocytes and interneurons and switch their fate to generate granular neurons, allowing the compensatory replenishment of these neurons via Hedgehog signaling following cerebellar injury. Whether IGPs are identical to BGLPs and NEPs remains unclear. These three progenitors show several differences in the types of neurons differentiated (i.e., granular neurons, MLIs, or both) and/or the triggers to enhance neural differentiation. While IGPs and NEPs, but not BGLPs, have the lineage potential to generate granular neurons, this granular neuron differentiation is enhanced in IGPs by inhibiting histone deacetylase (HDAC) 1 and 2 activities due to exposure to valproic acid (VPA) (Kamiya et al., 2023) and in NEPs by activating Hedge-hog signaling following cerebellar injury (Wojcinski et al., 2017).

Wojcinski et al. (2017) further reported a rare population of progenitor cells in the outer EGL that retain Sox2 expression and can differentiate into granular neurons following cerebellar injury. However, IGPs are distinguished from this progenitor by their Bergmann glia-like morphology and cerebellar cortical location and by serving at physiological conditions.

## 3 Possible cerebellar function and evolutionary significance of IGPs

The non-motor functions of the cerebellum are mediated by the hemispheres (or the neo-cerebellum), which exhibit morphological and functional asymmetry and have lateralized connections to other brain regions (Hu et al., 2008; Wang et al., 2013; Habas, 2021). The evolutionary expansion of the cerebellum is linked to the complexity of lobulation/sublobulation and the expansion of the cerebral cortex across mammalian species (Heuer et al., 2023; Carey, 2024). Volume lateralization and/or lobulation/sublobulation of the cerebellum are more pronounced in the order of mice, ferrets, and macaque monkeys (Sawada et al., 2015, 2020; Sawada and Saito, 2022). Cerebellar granular neurons are the most numerous type of neurons in the brain, estimated to number approximately 300 million in rodents and 45 billion in humans (Altman and Bayer, 1997a). Therefore, the evolutionary expansion of the cerebellum may have primarily depended on an increase in the number of granular neurons.

As mentioned above, IGPs may be another source of granular neurons distinct from EGPs in the developing cerebellum of ferret neonates (Kamiya et al., 2023). VPA acts on a natural source of neural progenitors in the developing brains, such as SVZ progenitors (Wang et al., 2016; Sawada, 2022) and hippocampal dentate gyrus progenitors (Fujimura et al., 2016; Sawada et al., 2021), to modulate their proliferation and neuronal differentiation by inhibiting HDAC 1 and 2 activities. In the cerebellum of ferret neonates, VPA stimulates the differentiative division of IGPs towards granular neurons, but reduces the proliferation of EGPs, suggesting an epigenetic regulation of the balance of granular neuron numbers arising from two distinct origins. Granular neurogenesis from IGPs may be involved in evolutionary expansion, structural and functional asymmetry, and morphological complexity (lobulation/sublobulation), forming individual differences, relevant to the acquisition of cerebellar non-motor functions during mammalian evolution. However, the existence and number of IGPs in the cerebellum of mammals other than ferrets, such as mice, monkeys, and humans, are unknown. This issue must be addressed in future studies.

## 4 Conclusion

This paper highlights a neural progenitor line that appears in the developing cerebellar cortex, termed “IGP,” found in ferrets and its potential as another source of granular neurons distinct from EGPs. IGPs may be a subpopulation of immature Bergmann glia as they have immature Bergmann glia-like characteristics, such as Sox2 expression and an extension of GFAP-positive radial processes throughout the ML to the EGL, despite expressing Nestin (Figures 1A, B). Conversely, whether IGPs are identical to known neural progenitors, such as the BGLPs reported by Suyama et al. (2026) and NEPs reported by Wojcinski et al. (2017), remains unclear. Neonatal exposure to VPA facilitates the transition of IGPs, rather than EGPs, into granular neurons through differentiative division (Kamiya and Sawada, 2023; Kamiya et al., 2024). Granular neurons provide excitatory mossy fiber input to Purkinje cells, which are inhibitory modulated by MLIs (Consalez et al., 2021). Accordingly, the excess production of IGP-derived granular neurons following neonatal VPA exposure may disrupt the balance between the excitatory and inhibitory inputs that regulate Purkinje cell activity, indicating developmental alterations relevant to neurodevelopmental disorders. Neonatal VPA exposure has been reported to induce autism spectrum disorder-like social behavioral deficits in ferrets (Krahe et al., 2016). These findings indicate that the abnormal development of IGPs relates to neurodevelopmental disorders associated with non-motor cerebellar function, such as autism spectrum disorder, and support our hypothesis that IGP-derived granular neurons play a role in non-motor cerebellar functions, such as language, cognition, and emotion. Further research on IGPs is expected to provide new perspectives for the investigation of cerebellar corticohistogensis, particularly the differences between motor and non-motor cerebellar regions, and how the abnormal development of IGPs relates to neurodevelopmental disorders.

## Data Availability

The original contributions presented in the study are included in the article/supplementary material, further inquiries can be directed to the corresponding author.
